# Telehealth mental health services during COVID-19: summary of evidence and clinical practice

**DOI:** 10.1177/1039856220943032

**Published:** 2020-07-28

**Authors:** Rebecca E Reay, Jeffrey CL Looi, Philip Keightley

**Affiliations:** Academic Unit of Psychiatry and Addiction Medicine, Australian National University Medical School; and ACT Health, Australia; Academic Unit of Psychiatry and Addiction Medicine, Australian National University Medical School; and ACT Health, Australia; Academic Unit of Psychiatry and Addiction Medicine, Australian National University Medical School; and ACT Health, Australia

**Keywords:** Telehealth, telemental health, telepsychiatry, videoconferencing, clinician-guided

## Abstract

**Objective:**

To provide a rapid clinical update on the evidence for telehealth in mental healthcare in the context of the COVID-19 pandemic public health measures.

**Conclusions:**

Telehealth has been rapidly implemented in metropolitan and rural settings and the existing evidence base demonstrates that it represents an effective mode of service delivery.

The outbreak of the COVID-19 pandemic has resulted in a major transformation in the way mental health interventions are delivered in Australia.^1^ Mental health clinicians and services had to rapidly transition to largely telehealth delivery to ensure continuity of care to patients and families. Private psychiatrists and allied health practitioners have made a rapid transition to the provision of healthcare via telehealth,^2,3^ assisted by new Medicare items. These changes have the potential to extend the reach and accessibility of mental healthcare to all Australians. On the other hand, clinicians with a strong preference for in-person treatments and those without experience in technology-delivered interventions, may find the transition challenging. It is timely to consider some of the issues around the use of telehealth in mental healthcare, including its effectiveness for common mental health conditions, advantages and disadvantages and recommendations for its implementation and evaluation.

Telehealth has been described as ‘the next big frontier in the efficient and effective delivery of health care’ (p. 621).^4^ Defined as the delivery of psychological and mental health services via telecommunication technologies, modalities include telephone-delivered therapy, videoconferencing, mental health apps and internet-delivered programs.^5^ Furthermore, telehealth interventions can be described as synchronous or asynchronous.^6^ Synchronous treatment is interactive communication that occurs in real time, such as telephone and video conferencing, and is the most similar to face-to-face treatment. Asynchronous treatments include emails, text, faxes, apps and online programmes. Many practitioners already use asynchronous interventions to check on patient progress, provide supplementary materials, online assessments and recommend mental health apps or online programs. Effectiveness of phone and videoconferencing-delivered interventions.

Research into telemental health provided to children, young people and adults demonstrates that interventions are feasible, acceptable, and as effective as in-person services.^7,8^ For example, a recent meta-analysis of synchronous telehealth treatments reported on its effectiveness for adults (including veteran populations) with common mental health disorders (e.g. depression, anxiety, posttraumatic stress disorder and adjustment disorder).^4^ The majority of studies consistently found telephone or videoconferencing therapy was as effective as standard in-person treatment and superior to treatment as usual. Both interventions showed clear, consistent evidence of beneficial effects. Furthermore, the generalisability and applicability of the results were rated as moderate to high. On the other hand, the evidence base for internet-delivered text-based therapy (via webchat) was promising but inconclusive.

## Effectiveness of internet-delivered interventions (self-guided versus clinician-guided)

Over three decades of research has provided substantial evidence for the effectiveness and acceptability of internet-delivered treatments delivered to children, adolescents^9^ and adults.^4^ Treatment often consists of online lessons, printable summaries, homework assignments, email reminders and resources. The most widely studied is cognitive behaviour therapy (iCBT),^10^ with strong evidence that it is as effective as clinician-delivered CBT and for a wide range of mental and physical health conditions.^9,10^ In contrast, a systematic review found that there was no substantive evidence iCBT was equally beneficial to in-person CBT for anxiety disorders.^11^ There is good evidence for the efficacy of online-delivered interpersonal psychotherapy,^12^ acceptance and commitment therapy^13^ and psychodynamic approaches.^14^ Although these approaches can be self-guided, *therapist support makes a substantial difference in terms of adherence, completion and efficacy*.^15^ This assistance can include regular texts, emails, private forums, phone, videoconferencing or in-person sessions. Furthermore, there is growing evidence indicating that ‘second-generation’ self-guided treatments, with enhanced engagement features, can produce clinical benefits similar to clinician-guided treatments.^16,17^ There has been a call to integrate internet-delivered services with traditional mental health services using a stepped care model. Clients identified as being suitable for online interventions could be directed to self-guided or therapist-guided programs, whilst those deemed unsuitable, or have not responded, are provided with face-to-face interventions^15^ or a hybrid approach.^18^ The Department of Health ‘Head to Health’ website provides an easy portal and guide to a wide range of evidence-based online programmes for consumers and providers.^19^

## Attitudes towards telehealth

A systematic review into clinician satisfaction with telehealth in mental healthcare (using videoconferencing) showed overall attitudes were largely positive.^20^ The findings were observed across different populations, locations (e.g. home, schools, crisis centres) and types of services (psychotherapy, assessment and medication management). Therapists reported telehealth was an effective, useful and acceptable way to deliver treatment. The majority of participants who received internet-delivered interventions report they are satisfied or very satisfied, describing advantages, such as its accessibility, convenience, low cost and greater privacy.^15^ We have heard anecdotal reports that telehealth sessions have significantly reduced the number of missed appointments and dropouts, potentially increasing treatment adherence and the efficiency of mental health services. However, telehealth may be less useful in patients with significant social disadvantage, and severe mental illnesses that impair cognitive abilities and insight such as schizophrenia and major neurocognitive disorder; further research for this population is needed.^21^

### Addressing barriers to telehealth

Clinicians largely report positive attitudes towards telehealth approaches, and attitudes have been shown to improve with use, for both clients and clinicians.^22^ Clinicians have expressed concerns about its impact on rapport building, the therapeutic relationship, privacy and safety issues. The reduced non-verbal communications (e.g. inflection, tone, gestures and mannerisms) can be a deterrent for some. Some therapists are also of the view that it is less effective than in-person therapy and lack experience or interest in technology-delivered interventions. Previously reported concerns about the extra hassle, frustration and technological limitations may be rapidly outdated in a new environment where the technology has suddenly and necessarily become more familiar. Given clinicians often serve as ‘gatekeepers’ for its implementation,^23^ services must address their concerns in order to improve its acceptance and sustainability. Such an approach should provide guidance on best practice, developing the therapeutic relationship, and innovative ways to deliver traditional treatments. Training should also increase clinicians’ comfort and experience with the new technologies, whilst ensuring responsive technical support. Importantly, there are some for whom internet-delivered treatment may not be suitable, including older persons and those with significant disadvantage. Providers need to assess each individual’s suitability, including their capacity to access technology, severity of symptoms, presence of psychosis or risks of harm.

## Telehealth evaluation

Various models for telehealth evaluation have been proposed in the Australasian setting. Different stakeholders may have very different outcome priorities. For example, a psychiatric hospital may seek earlier discharge and avoidance of readmission, a patient may be focused on usability and convenience, and a health service may be concerned by the time and workforce needed to set up the technology and availability of scarce equipment. Four helpful dimensions for evaluation include patient control, clinician quality of care, organisation sustainability and technology capability or capacity.^24^ These should be linked to robust measures that are specific to the situation or setting in which the technology is used. Further research is needed on the acceptance, adherence and effectiveness of telehealth for mental healthcare in real-world settings, particularly with patients who have complex and severe mental illness.

## Conclusions and future directions

As telehealth becomes more commonly used in mental healthcare, it will be important to evaluate its relative outcomes and effectiveness. Mental health professionals have different skill sets, and research into effectiveness of telehealth will also need to be targeted within professions. Accordingly, developments of specific professional training and competencies for provision of care will be needed. Telehealth technology also needs ongoing research, given the challenges to cybersecurity that have been noted.^2^ Telehealth is complementary to existing care and thus evidence is needed into how it may integrate with face-to-face mental healthcare as well as other digital mental health services.
